# Supplementary material for: Reversible ionic aggregation kinetics in concentrated electrolytes
Source: Chem Sci. 2026 May 12;17(26):13171–85. doi: 10.1039/d6sc01986b (PMC13216889; doi:10.1039/d6sc01986b)
Supplement: SC-017-D6SC01986B-s001 [file SC-017-D6SC01986B-s001.pdf]

Supplementary Information (SI) for Chemical Science.
This journal is © The Royal Society of Chemistry 2026

Supporting Information: Reversible Ionic Aggregation Kinetics in Concentrated Electrolytes

Zachary A. H. Goodwin

John A. Paulson School of Engineering and Applied Sciences, Harvard University, Cambridge, Massachusetts 02138, United States

Department of Materials, University of Oxford, Parks Road, Oxford OX1 3PH, United Kingdom and

zac.goodwin@materials.ox.ac.uk

(Dated: May 15, 2026)

I. ION PAIR KINETICS

The notation here follows that of the main text. For brevity, we will not repeat definitions. Let us study a simple case of reversible ion pairing, described by the equilibrium

$$\mathrm{c}_{10} + \mathrm{c}_{01} \underset{\mathrm{b}}{\overset{\mathrm{a}}{\rightleftharpoons}} \mathrm{c}_{11}. \tag{S1}$$

We know the concentrations of free cations is given by

$$c_{10} = c_+(1 - p_{+-}), \tag{S2}$$

and free anions

$$c_{01} = c_-(1 - p_{-+}), \tag{S3}$$

and the concentration of ion pairs is determined from

$$c_{11} = \lambda c_{10} c_{01}, \tag{S4}$$

where the association constant is related to the rates through

$$\lambda = \frac{a}{b}. \tag{S5}$$

We have statements of incompressibility through

$$c_+ + c_- = 1, \tag{S6}$$

and also

$$2c_{11} + c_{10} + c_{01} = 1. \tag{S7}$$

Since it is a symmetric system, $p_{+-} = p_{-+} = p(t)$ and $c_+ = c_- = c_\pm = 1/2$, let us consider the rate of change of free cations

$$\frac{dc_{10}}{dt} = -ac_{10}c_{01} + bc_{11} \tag{S8}$$

$$\frac{dc_{10}}{dt} = -ac_{10}c_{01} + \frac{b}{2}(1 - c_{10} - c_{01}). \tag{S9}$$

From using Eqs.(S2),(S3),(S7), we can arrive at

$$\frac{d[c_\pm(1-p)]}{dt} = -a[c_\pm(1-p)]^2 + \frac{b}{2}[1-2c_\pm(1-p)], \tag{S10}$$

which simplifies to

$$\frac{dp}{dt} = \frac{a}{2}(1-p)^2 - bp, \tag{S11}$$

which can be obtained as a limit from the equation in the main text.

II. ASYMMETRIC CASE

Having established the equations for the simplest, symmetric case in the main text, in this section we extend the solutions to $\psi_+ \neq \psi_-$, referred to as the asymmetric case. Fundamentally, the governing equations are the same form, but the solution is slightly more involved since there are two probabilities, p_{+-} and p_{-+}.

As demonstrated in Ref. 1, the time-dependence of the association probabilities is governed by the macroscopic rate equation, give by

$$\frac{d[A^R]}{dt} = k[A^F][B^F] - f[A^R], \tag{S12}$$

where $[B^F]$ is again a concentration of free association sites [1]. Here we take A to correspond to alkali metal cation sites that can bind to anions, and B denotes anion sites that can bind to alkali metal cations. Therefore, our governing equation for the change in p_{+-} is given by [1]

$$\frac{1}{k}\frac{dp_{+-}}{dt} = \psi_-(1-p_{-+})(1-p_{+-}) - \frac{p_{+-}}{\lambda}. \tag{S13}$$

Clearly, at equilibrium we arrive at

$$\psi_-\lambda = \frac{p_{+-}}{(1-p_{-+})(1-p_{+-})}, \tag{S14}$$

which is the mass action law in the main text. We can solve Eq. (S13), with the initial condition $p_{+-}(t=0) = p^0_{+-}$, and arrive at

$$p_{+-} = \frac{p^\infty_{+-}(1-\Lambda_\pm e^{-t/\tau_\pm})}{1-p^\infty_{+-}p^\infty_{-+}\Lambda_\pm e^{-t/\tau_\pm}}, \tag{S15}$$

where we have defined

$$\Lambda_\pm = \frac{p^\infty_{+-} - p^0_{+-}}{p^\infty_{+-}(1-p^\infty_{-+}p^0_{+-})}, \tag{S16}$$

and the inverse relaxation time is given by

$$\tau_\pm^{-1} = \frac{k}{\lambda}\sqrt{[1+\lambda\psi_\pm]^2 - 4\lambda^2\psi_-\psi_+} \tag{S17}$$

$$= k\psi_+\frac{1-p_{+-}p_{-+}}{p_{-+}}, \tag{S18}$$

This solution is analogous to equation for $p(t)$ in the main text, and recovers it in the limit of the symmetric case [1].

Equivalently, we could write the counterpart of Eq. (S12), and arrive at the governing equation in terms of p_{-+}

$$\frac{1}{k}\frac{dp_{-+}}{dt} = \psi_{+}(1 - p_{-+})(1 - p_{+-}) - \frac{p_{-+}}{\lambda}, \tag{S19}$$

which also recovers the mass action law at equilibrium, and yields, with the initial condition p^0_{-+}, the following solution

$$p_{-+} = \frac{p^{\infty}_{-+}(1 - \Lambda_{\mp} e^{-t/\tau_{\pm}})}{1 - p^{\infty}_{+-} p^{\infty}_{-+} \Lambda_{\mp} e^{-t/\tau_{\pm}}}, \tag{S20}$$

where we have defined

$$\Lambda_{\mp} = \frac{p^{\infty}_{-+} - p^{0}_{-+}}{p^{\infty}_{-+}(1 - p^{\infty}_{+-} p^{0}_{-+})}, \tag{S21}$$

with the relaxation time again being given by Eq. (S18). Finally, we note that one can convert between Eq. (S15) and Eq. (S20) through a time-generalized conservation of associations.

In the post-gel regime, these equations still apply, but the total association probabilities are decomposed into sol and gel contributions, using Flory's post-gel treatment, as described in the symmetric case [1]. We note that, also as discussed in the symmetric case, we can take limits of irreversible aggregation and fragmentation, where simplified solutions would also be found for the asymmetric case.

III. SUPPORTING RESULTS

A. Symmetric case

In Fig. S1, we show the radial distribution function, averaged over 10 time steps, at the indicated average times. The first peak in $g(r)$ increases over time, but even at small times, there is a clear peak at ∼2.5 Åwhich corresponds to the ionic aggregates. We choose the real space cutoff to count associations to be approximately where the $g(r)$ reaches 1 again after the first maximum. This turns out to be ∼3.3 Å, which is indicated in Fig. S1 by the vertical line. Other real space cutoffs were investigated, such as ∼3 Åand ∼3.65 Å(the first minimum after the first maximum), but all this only changes the exact values of the coordination numbers, not the qualitative results.

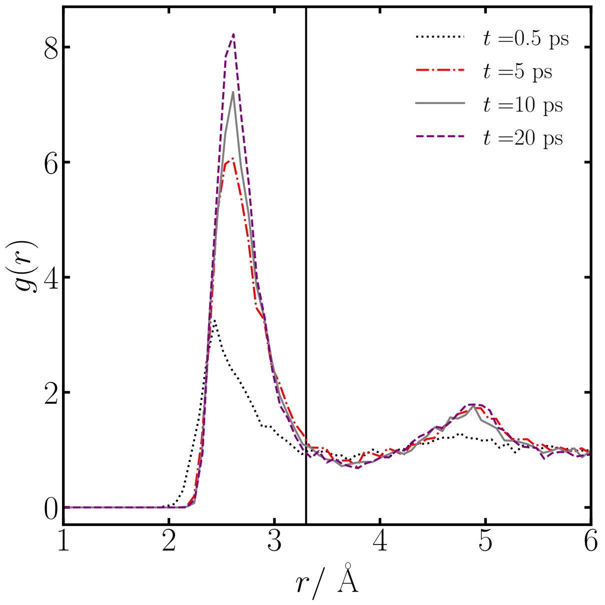

FIG. S1. Radial distribution function computed over 10 time steps, for various times as indicated. The vertical line denotes where we choose to define the cutoff distance for an association.

In Fig. S2, we show probability distributions of the different coordination numbers of $TFSI^-$ bound to Na^+ for several times (0.1, 0.5 and 1 ps, in left, middle, right, respectively). We can see that the distributions remain to be roughly normal through time. Moreover, we see that there is only a few examples of 5 $TFSI^-$ anions coordinated to Na^+, which motivates the use of $f_+ = 4$. For the $TFSI^-$ anions, $f_- = 3$ is known to be a good value [2, 3].

In Fig. S3, we display the changes in coordination numbers as a function of time for

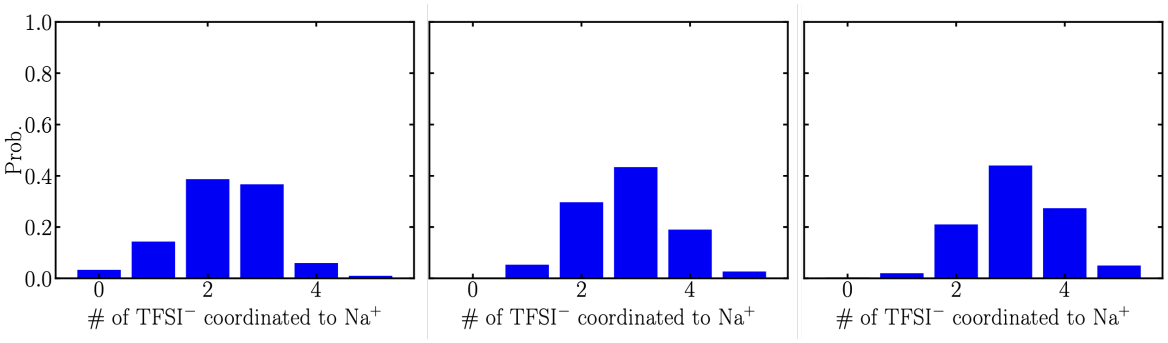

FIG. S2. Probability distribution of different numbers of TFSI$^-$ anions bound to Na$^+$ at various times 0.1, 0.5 and 1 ps in left, middle, right, respectively, for the symmetric case at 300 K.

the step-change in temperature condition. Initially the simulation was at 300 K, and the final temperature is indicated. Note that the coordination numbers start at larger values than those at the end of the runs in the main text, but this could be attributed to the simulations being at 300 K while at the density of the indicated temperature, and from the longer equilibration times. For both cases, the coordination numbers decrease slightly with time, with the larger temperature change resulting in a larger reduction in coordination numbers. We find qualitatively similar results as the main text. Specifically, the theory underestimates the change at small times, but overestimates the change at longer times. The disagreement is not as strong as the main text, but there are only modest changes in coordination numbers from the step-change in temperature.

In Fig. S4(top) we show the autocorrelation function of associations persisting as a function of time. Details of this calculation is provided in the Molecular Dynamics Methods Section. It is evident that this cannot be fitted by a single exponential function. At small times ($\sim$1 ps), there is an initial rapid exponential decrease in this measure, as seen in the right panel. After which, the decay transitions to a slower decay. In Fig. S4(bottom) we fit these functions to provide the persistence/residence times of the associations, i.e., the rate of breaking associations if we assume irreversible aggregation. The respective association breaking decay rates are reported in the main text. These observations are consistent with those of other electrolytes, such as ILs, WiSEs, SiILs and battery electrolytes.

In Fig. S5, we show the fits for the theory over a short (a) and long (b) time scale. For the short time fit in Fig. S5a), we find significantly better agreement than the main text. The theory still slightly underestimates the coordination numbers at short times and

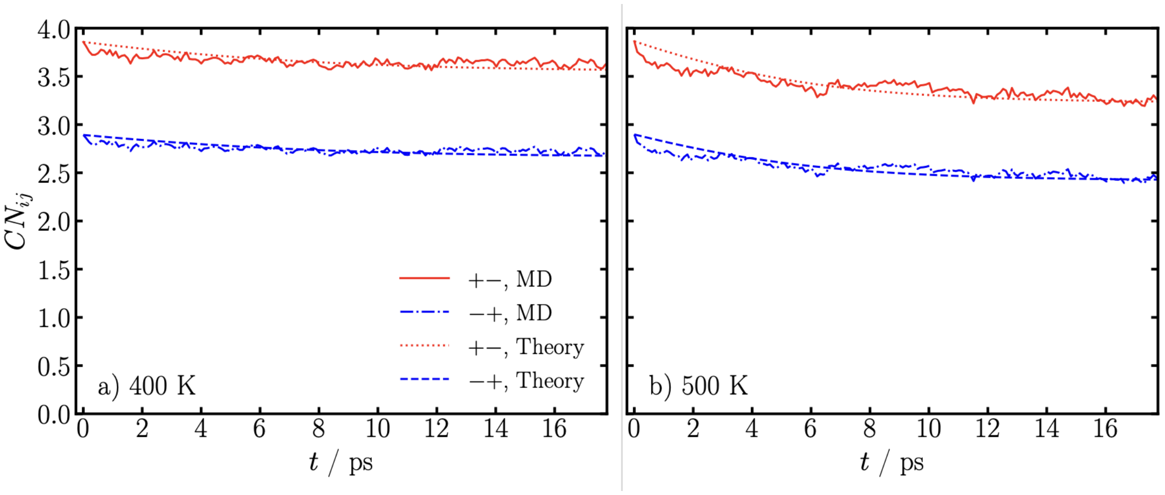

FIG. S3. Coordination numbers as a function of time for the temperature step-change in conditions at the indicated temperatures. Initially the simulations were at 300 K and they were step-changed to the indicated temperatures.

overestimates at long, but the fit is within the fluctuations in the coordination numbers. The fitted inverse time constant was $\tau^{-1} = 1$ ps^{-1}, which is larger than those in the main text, indicating a smaller time scale. In the long time fit in Fig. S5b), similar agreement is found to the main text. A slight increase in coordination numbers is observed over the time scales in the main text, but it is harder to observe the discrepancy between the theory and simulation at short times.

In Fig. S5c) and d), we also show a comparison of the parameter-free models, based on extracting the rate of forming an association from Fig.Fig. S4(bottom). For the short-time case, slightly worse agreement is found in comparison to Fig. S5a), but the fit is still satisfactory. Whereas for the long-time case, the parameter-free theory does not reproduce the overall curve. This is because the short-time changes in coordination numbers dominate, and there is a memory effect from the non-equilibrium conditions, which means the long-time rate of forming associations is drastically off. Despite this, the slope of CN_{ij} at long times in Fig. S5d) does appear to match the simulated slope. This is promising for the developments to a quantitative theory, based on rate kernles which change in time using these extracted rates.

In Fig. S6 we show the cluster bond density (CBD) as a function of the number of ions in an aggregate at the indicated times, for the symmetric case of the main text. The cluster

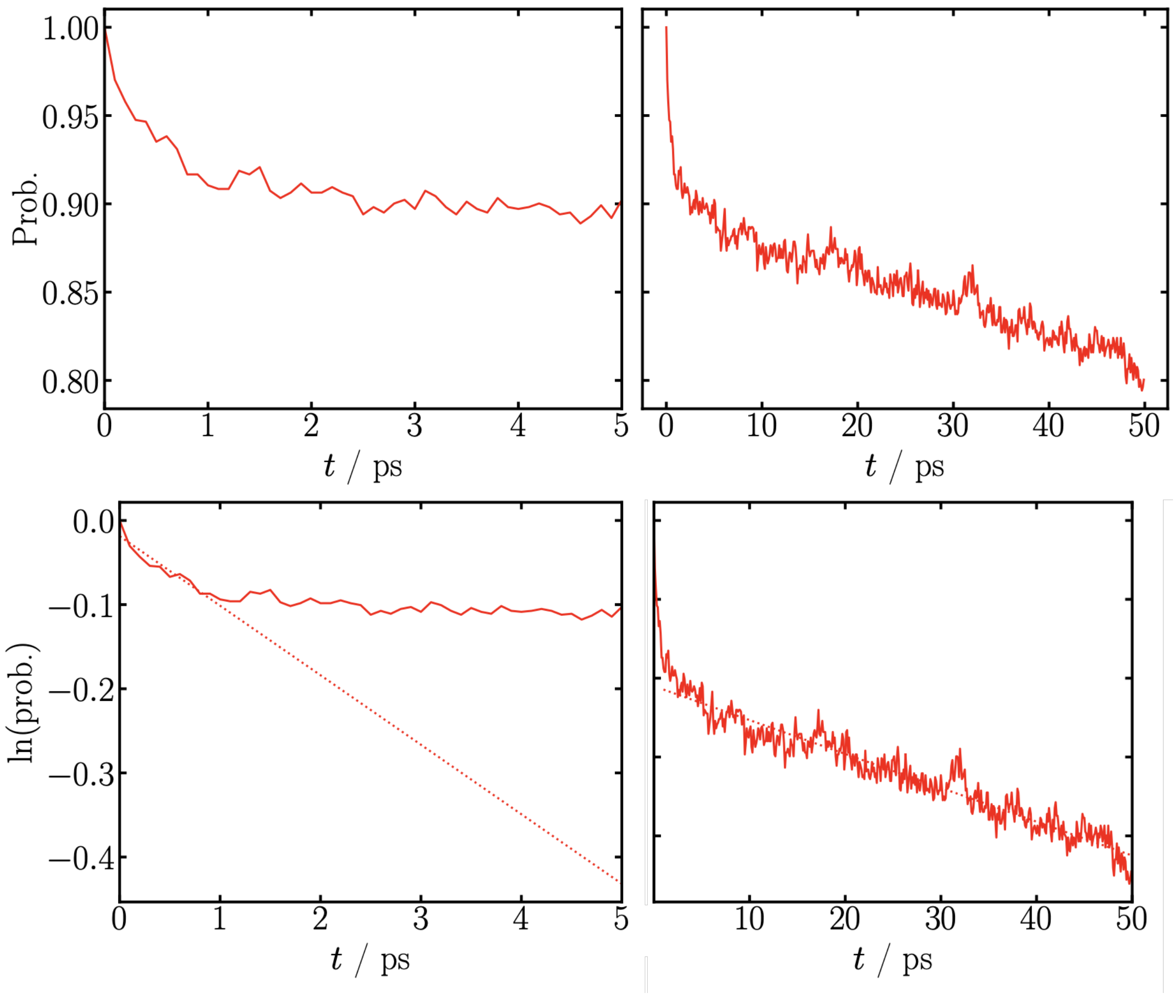

FIG. S4. (top) - Autocorrelation function of associations existing as a function of time, for a long time scale (left), and a zoom in of the short initial time (right). (bottom) - log of the Autocorrelation function of associations existing as a function of time to extract the decay rate at short (left) and long (right) times, which directly provides f, assuming the exponential decay of irreversible aggregation applies.

bond density is calculated from the number of associations in an aggregate over the number of ions in that aggregate. In the Cayley tree limit, we have $(l+m-1)/(l+m)$, which never exceeds 1. If there are loops, closed paths of ionic associations, the CBD can be larger than 1. In the studied cases, there is only a few examples of aggregates with loops. The 4 ps case has a increased the number of loops, suggesting loop formation takes some time. The bulk equilibrated results at low mole fractions are known to have many loops, which further supports this observation [3]. This could be a reason why there is good agreement with the

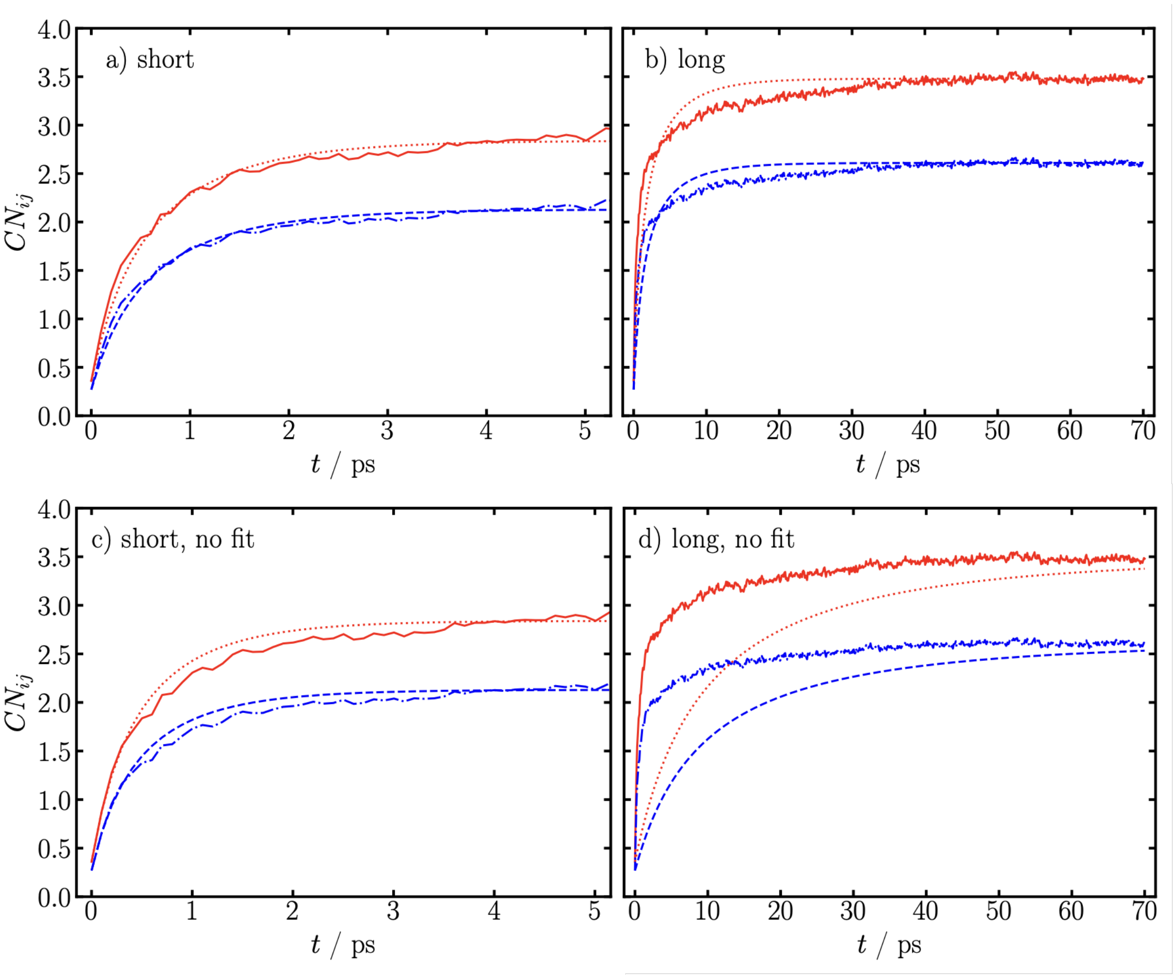

FIG. S5. Coordination numbers as a function of time for the charge rescaling step-change in conditions at 300 K. a) for the short time fit b) the long time fit. c) for the case of short time, with the rate extracted from Fig. S4 d) for the case of long time, with the rate extracted from Fig. S4.

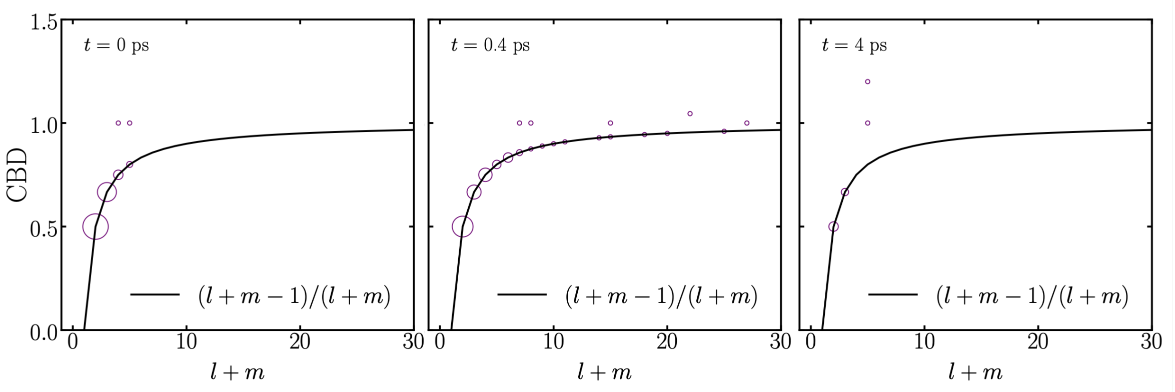

FIG. S6. Cluster bond density as a function of cluster size at the indicated times.

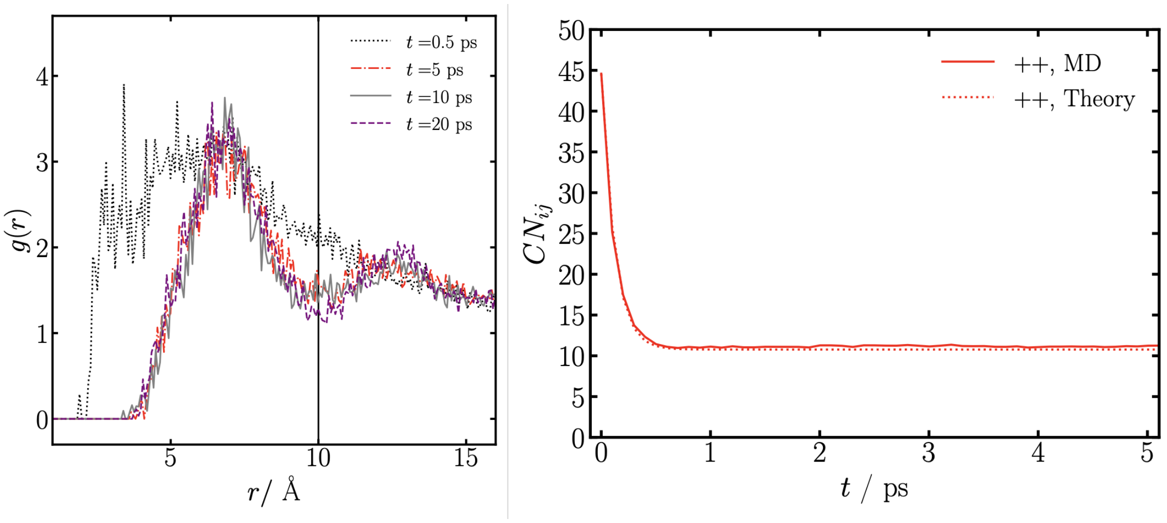

FIG. S7. Pair correlation function between cation-cation (left) and how the coordination numbers of cation-cation change as a function of time (right).

gel time from the association probabilities and the changes in the cluster distribution.

In Fig. S7(left), we show how the pair-correlation function between cation-cation changes with time. Initially, in the absence of the charges, Na^+-Na^+ can approach to 3 Å, with a broad, flat peak around 5 Å. Upon turning on the interactions, the peak shifts towards 7 Åwithout growing in magnitude, with the first pronounced minimum occurring at 10 Å. Sticking with the real-space cutoff to define correlations (associations) between Na^+-Na^+, we have cutoff distance of 10 Å.

In Fig. S7(right), we show how the corresponding Na^+-Na^+ coordination numbers from this cutoff change with time. To obtain probabilities between 0-1, a functionality (f_+) of 45 is required, which is clearly not a physical value, and reflects the Na^+-Na^+ "associations" not being a good approximation, justifying their neglect in the main text. Upon switching on the interactions, the coordination number quickly decreases to ∼11 in ∼0.5 ps. A fitted version of the theory (just based on cation-cation interactions, symmetric case) well reproduces these changes with a single rate constant.

Similarly in Fig. S8(left), we show how the pair-correlation function between anion-anion (taken between the O atoms in different anions) changes with time. Initially, there is a small peak at ∼3.5 Å, and a first minimum at ∼4.3 Å, which is taken to be the real-space cutoff for the anion-anion correlations (associations). With increasing time, there are modest changes to this structure. This is in contrast to the large changes in cation-anion correlations with

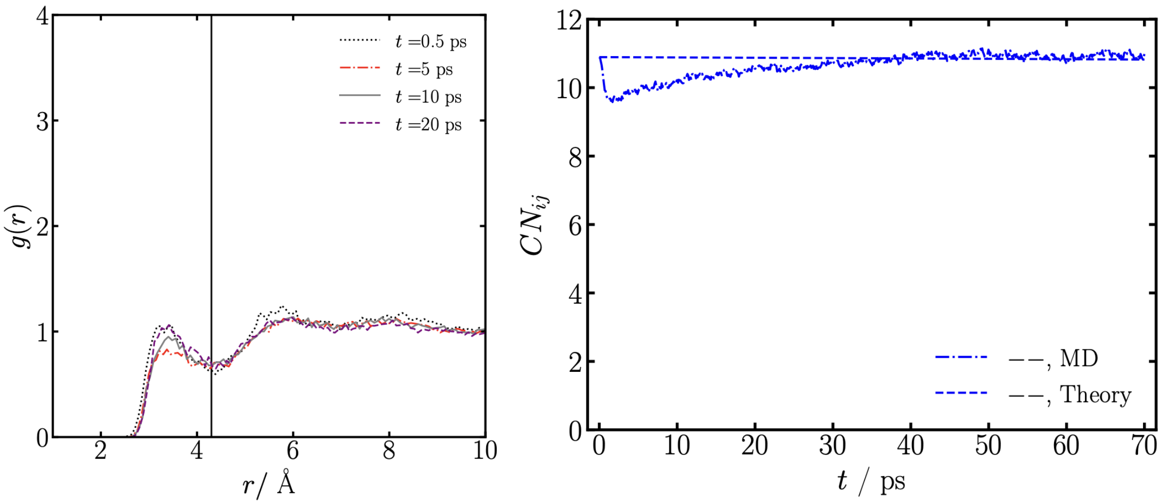

FIG. S8. Pair correlation function between anion-anion (left) and how the coordination numbers of anion-anion change as a function of time (right).

time, further supporting only retaining the interactions considered in the main text.

In Fig. S7(right), we show how the corresponding anion-anion coordination numbers from this cutoff change with time. Again to obtain probabilities within 0-1, a functionality (f_-) of 12 is required, which is not a physical case, and reflects treating these correlations as associations as questionable. Initially, after turning back on the interactions, we find there is a small, sudden drop in the anion-anion "coordination numbers", before it slowly recovers over 40 ps and increases slightly over its initial value. This suggests that the dynamics of anion-anion interactions is the source of the multiple time scales observed in the main text.

B. Asymmetric case

In this section we further test the asymmetric case of the equations, which come from studying other mole fractions than those in the previous section. Analogous observations are found to the symmetric case, but it further cements those findings.

In Fig. S9 we show how CN_{ij} changes with time, again from the charge rescaling non-equilibrium simulations, for the indicated mole fractions x in $\text{NaTFSI}_x\text{EMIMTFSI}_{1-x}$ at 300 K. For the lowest mole fraction case, $x = 0.25$, as seen in Fig. S9a), using the initial and final probabilities from MD, we fitted a decay time of ~0.52 ps^{-1}, and a rate constant of forming an association of ~2.2 ps^{-1}. Overall, the theory again does a reasonable job at capturing the trends in the simulations, although it again underestimates the coordination numbers at short times and overestimate them at long times. Very similar observations are found for the mole fractions of $x = 0.5$ and $x = 2/3$, as shown in Fig. S9b)&c), respectively.

In Tab. S1, we report the fitted parameters of our model. With increasing mole fraction of Na-salt, the association constant increases substantially, which is consistent with the expectation of IL cations interacting with the TFSI^- anions [4]. We find the inverse time scale τ^{-1} decreases with increasing mole fraction of Na-salt, which indicates the time scale increases with increasing mole fraction of Na-salt. Since the associations are becoming stronger, this intuitively makes sense. We find the rate of forming associations increases with increasing mole fraction of Na-salt, suggesting it is becoming easier to form associations in more Na-rich environments.

For the $x = 0.25$ and $x = 0.5$ mole fractions, we predict the critical times of gelation in Fig. S10. For the $x = 0.25$ case, as shown in Fig. S10a), the electrolyte at long times only just resides above the critical condition to form a percolating ionic network. We find that at ~4 ps, both MD simulations and theory predict the transition into the gel regime. Whereas, for the $x = 0.5$ case, as shown in Fig. S10b), the electrolyte is well in the gel regime. The MD simulations predict a critical gel time at ~0.75 ps, while the theory lags behind these simulations at ~1 ps.

In Fig. S11, we plot the calculated cluster distributions from MD simulations and theory at various times for the mole fractions $x = 0.25$ and $x = 0.5$. In the top rows, which corresponds to $x = 0.25$, free ions initially ($t = 0$) dominate the simulations, with a small concentration of aggregates containing fewer than 5 ions. At $t = 6$ ps, which should corre-

x	τ^{-1} / ps^{-1}	k / ps^{-1}	λ
1/4	0.517	2.223	23.916
1/2	0.478	2.535	34.322
2/3	0.403	3.214	54.736

TABLE S1. Summary of inverse time scales (τ^{-1}), rate of forming an association (k), and association constant (λ) for the studied compositions of the asymmetric case.

spond to the electrolyte having entered the gel regime, we find aggregates containing up to 20 ions, but with free anions still dominating the distribution and no free cations existing. In addition, we find a percolating ionic network in the simulation. At a later time, $t = 15$ ps, we find practically the same cluster distribution. In Fig. S10, $p_{+-}p_{-+}$ does not grow substantially over the critical point, so the changes in the cluster distribution follow well these predictions form the critical gel criteria.

Whereas, for $x = 0.5$, as shown the bottom row of Fig. S11, we find results more similar to the symmetric case previously described. At $t = 0.8$ ps, which is a time just larger the predicted critical gel time in Fig. S10b), we find a large distribution of aggregates, containing up to 30 ions, in addition to a percolating ionic network. At a later time of $t = 4$ ps, the cluster distribution is again diminished, with practically only free anions and no free cations.

Therefore, the cluster distributions further corroborate the predicted critical gel times from the association probabilities. Furthermore, this also supports the idea that the central variable is the association probabilities, which changes in time, and is used in the cluster distribution in a quasi-equilibrium way, with the theory predictions qualitatively matching the simulated values.

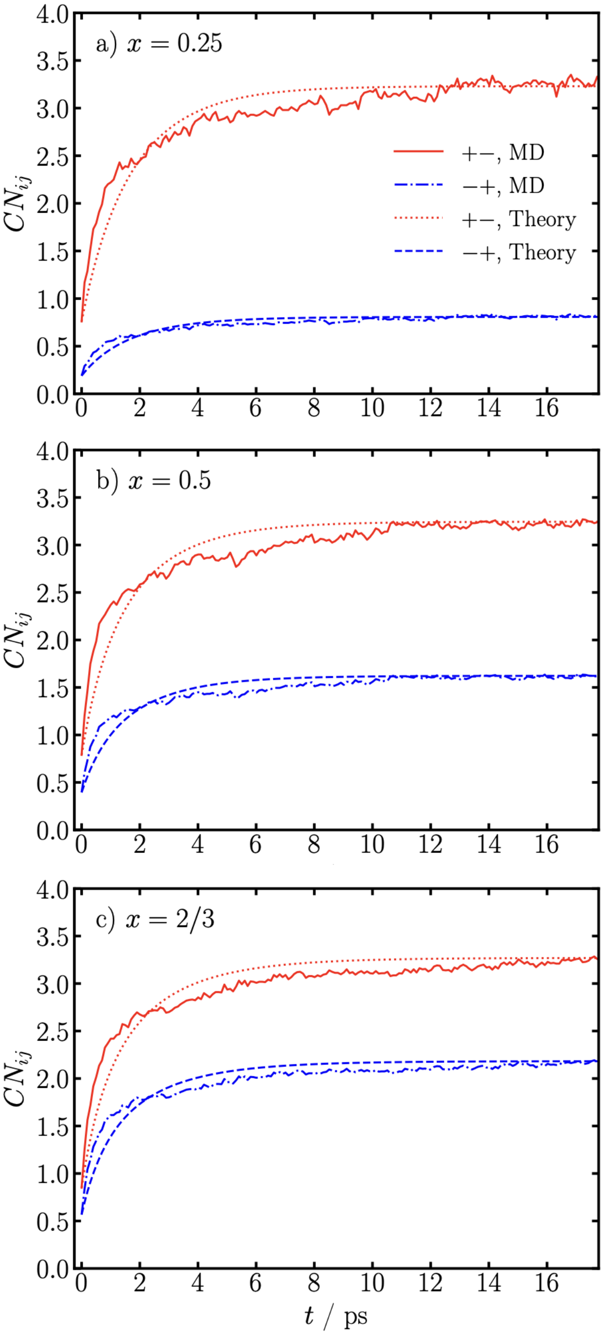

FIG. S9. Coordination numbers between Na^+ and $TFSI^-$ as a function of time, for the charge rescaling case, for the indicated mole fractions, in $NaTFSI_xEMIMTFSI_{1-x}$, at 300 K, for MD simulations and theory.

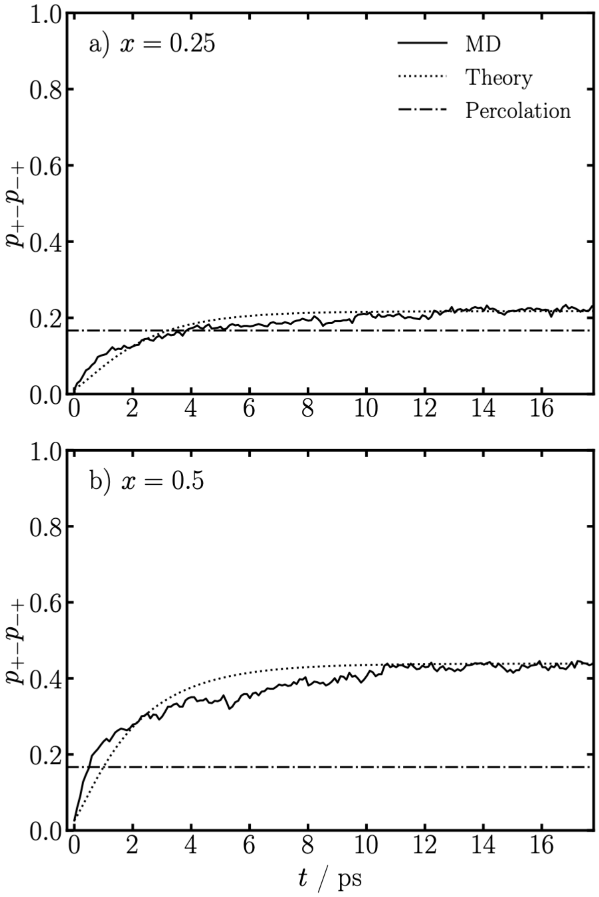

FIG. S10. Gelation criteria in as a function of time for different alkali metal mole fractions in NaTFSI$_x$EMIMTFSI$_{1-x}$, for the charge rescaling case, at 300 K, for MD simulations and theory. The percolation point is given by $p_{+-}p_{-+} = [(f_+ - 1)(f_- - 1)]^{-1}$.

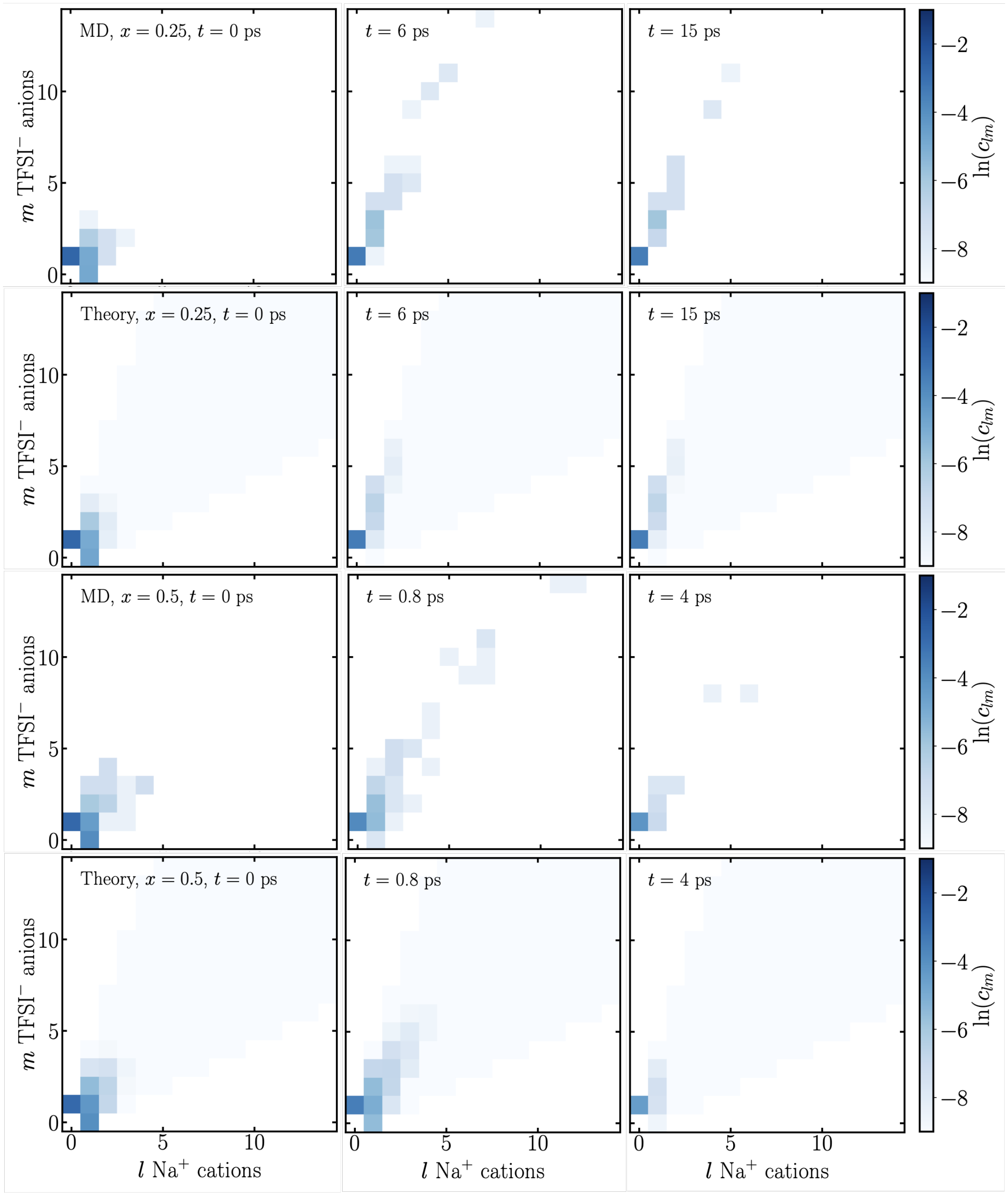

FIG. S11. Cluster distributions c_{lm}, with m TFSI$^-$ anions and l Na$^+$ cations in an aggregate, at various points in the MD simulations and theory, as indicated, at 300 K for the different alkali metal mole fractions in NaTFSI$_x$EMIMTFSI$_{1-x}$.

IV. DETAILS TO REPRODUCE GRAPHS

Here a brief description of how the graphs were produced will be provided.

To generate the non-equilibrium coordination number graphs:

1. From MD, the coordination numbers were computed as a function of time from ensemble averaging all of the ion environments at each time step

2. From the initial and final values of the coordination numbers, and the assumed functionalities, the initial and final association probabilities were extracted

3. Using these boundary conditions, and the analytically derived equations for how probabilities change with time, we fitted (unless otherwise stated) the inverse decay constant, τ^{-1}, for these curves over the time period shown

4. The theory is then determined, and figure with both simulations and theory included

To generate the non-equilibrium critical gel criteria:

1. From MD, the coordination numbers were computed as a function of time from ensemble averaging all of the ion environments at each time step

2. These MD coordination numbers were divided by their respective functionalities to obtain the corresponding association probabilities

3. Using the previous fitted τ^{-1}, and the initial and final association probabilities, the corresponding theory prediction was produced

4. The horizontal line indicates the critical criteria, which is simply determined from the functionalities of the ions

To generate the non-equilibrium cluster distribution:

1. From the MD simulations, adjacency matrices were constructed at each time to determine all of the connected ionic graphs

2. The dimensionless concentration of a cluster of rank c_{lm} from MD at each time step is then determined from N_{lm}, the number of clusters of that rank, divided by $\Omega = N_+ + \xi_- N_- + \xi_\oplus N_\oplus$

3. The corresponding theory graph is produced using how the theory association probabilities change in time to determine $\phi_{01/10}$ and from the mass action laws how the effective association constant changes in time

See data availability statement for more details where the scripts for these calculations, and additional scripts for the figures in the SI, can be found.

[1] P. G. J. van Dongen and M. H. Ernst, Journal of Statistical Physics **37**, 301 (1984).

[2] M. McEldrew, Z. A. Goodwin, S. Bi, A. Kornyshev, and M. Z. Bazant, J. Electrochem. Soc. **168**, 050514 (2021).

[3] X. Zhang, Z. A. Goodwin, A. G. Hoane, A. Deptula, D. M. Markiewitz, N. Molinari, Q. Zheng, H. Li, M. McEldrew, B. Kozinsky, *et al.*, ACS nano **18**, 34007 (2024).

[4] M. McEldrew, Z. A. H. Goodwin, N. Molinari, B. Kozinsky, A. A. Kornyshev, and M. Z. Bazant, J. Phys. Chem. B **125**, 13752 (2021).
